# Superresolution Imaging of Human Cytomegalovirus vMIA Localization in Sub-Mitochondrial Compartments

**DOI:** 10.3390/v6041612

**Published:** 2014-04-09

**Authors:** Shivaprasad Bhuvanendran, Kyle Salka, Kristin Rainey, Sen Chandra Sreetama, Elizabeth Williams, Margretha Leeker, Vidhya Prasad, Jonathan Boyd, George H. Patterson, Jyoti K. Jaiswal, Anamaris M. Colberg-Poley

**Affiliations:** 1 Research Center for Genetic Medicine, Children’s Research Institute, Children’s National Health System, 111 Michigan Avenue, NW, Washington, DC 20010, USA; E-Mails: SBhuvanendran@childrensnational.org (S.B.); kyle.salka@gmail.com (K.S.); Sreetama.SenChandra@childrensnational.org (S.C.S.); elizabeth.anne.williams.umd@gmail.com (E.W.); maggie.leeker@gmail.com (M.L.); VPrasad@childrensnational.org (V.P.); 2 Section on Biophotonics, National Institute of Biomedical Imaging and Bioengineering, National Institutes of Health, Bethesda, MD 20892, USA; E-Mail: kristin.rainey@nih.gov; 3 Life Science Division, Leica Microsystems, Inc., 1700 Leider Lane, Buffalo Grove, IL 60089, USA; E-Mail: Jonathan.Boyd@Leica-Microsystems.com; 4 Department of Integrative Systems Biology, George Washington University School of Medicine and Health Sciences, Washington, DC 20037, USA; 5 Department of Biochemistry and Molecular Medicine, George Washington University School of Medicine and Health Sciences, Washington, DC 20037, USA

**Keywords:** HCMV vMIA, MAM, mitochondria, OMM, matrix, confocal microscopy, superresolution microscopy, GSTED, MSIM, PALM

## Abstract

The human cytomegalovirus (HCMV) viral mitochondria-localized inhibitor of apoptosis (vMIA) protein, traffics to mitochondria-associated membranes (MAM), where the endoplasmic reticulum (ER) contacts the outer mitochondrial membrane (OMM). vMIA association with the MAM has not been visualized by imaging. Here, we have visualized this by using a combination of confocal and superresolution imaging. Deconvolution of confocal microscopy images shows vMIA localizes away from mitochondrial matrix at the Mitochondria-ER interface. By gated stimulated emission depletion (GSTED) imaging, we show that along this interface vMIA is distributed in clusters. Through multicolor, multifocal structured illumination microscopy (MSIM), we find vMIA clusters localize away from MitoTracker Red, indicating its OMM localization. GSTED and MSIM imaging show vMIA exists in clusters of ~100–150 nm, which is consistent with the cluster size determined by Photoactivated Localization Microscopy (PALM). With these diverse superresolution approaches, we have imaged the clustered distribution of vMIA at the OMM adjacent to the ER. Our findings directly compare the relative advantages of each of these superresolution imaging modalities for imaging components of the MAM and sub-mitochondrial compartments. These studies establish the ability of superresolution imaging to provide valuable insight into viral protein location, particularly in the sub-mitochondrial compartments, and into their clustered organization.

## 1. Introduction

**Figure 1:**
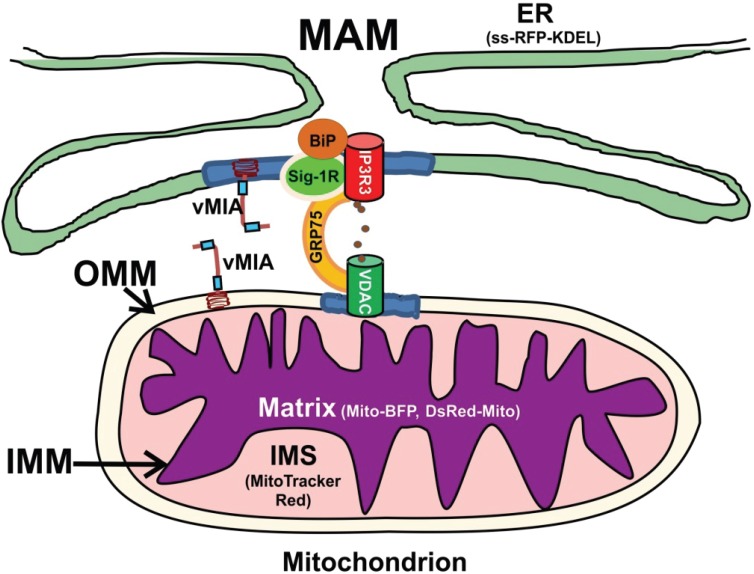
Endoplasmic reticulum (ER), mitochondria-associated membranes (MAM), and mitochondrial sub-compartments visualized. Viral mitochondria-localized inhibitor of apoptosis (vMIA) localization in the MAM and mitochondrion sub-compartments was imaged using the following markers: preprolactin signal sequence (ss) fused to the N-terminus of the red fluorescent protein (RFP) with a KDEL ER retention signal at its C-terminus (ss-RFP-KDEL, for ER) [36] and mitochondrial Cox 4 leader fused to Tag blue fluorescent protein (Mito-BFP)/mitochondrial targeting sequence from human cytochrome c oxidase subunit VIII to the N-terminus of *Discosoma* RFP (DsRed-Mito, for mitochondrial matrix) [37]. We use MitoTracker Red as an intermembrane space (IMS) marker based upon the superresolution imaging of MitoTracker by others [38,39] and our own results herein. Contacts between the ER and mitochondria are shown, with the MAM Ca^2+^ signaling complex components on the ER (IP3R3), cytosol (GRP75) and outer mitochondrial membrane (OMM) (voltage dependent anion channel (VDAC)). MAM Ca^2+^ efflux from the ER is regulated by chaperones (BiP, Sig-1R) as well as vMIA. Lipid rafts (blue) containing the Ca^2+^ signaling complex and vMIA are shown. These components are shown in the figure.

The mitochondria-associated membrane (MAM) sub-compartment of the endoplasmic reticulum (ER) plays critical roles in ER-mitochondrial cross-talk by allowing efficient transfer of calcium (Ca^2+^) from the ER to mitochondria without elevating cytosolic Ca^2+^ levels [1,2,3]. A macromolecular complex, composed of MAM inositol 1,4,5 trisphosphate receptors (IP3R), cytosolic glucose response protein 75 (GRP75) and outer mitochondrial membrane (OMM)-localized voltage dependent anion channel (VDAC) generates the high Ca^2+^ microdomains needed for Ca^2+^ transfer from ER to mitochondria (Figure 1) [4]. Constitutive low level IP3R-mediated Ca^2+^ transfer, needed for Ca^2+^ dependent mitochondrial enzymes, maintains normal cellular metabolism [5]. Nonetheless, continued mitochondrial Ca^2+^ influx drives the adaptive metabolic phase of early ER stress and can result in mitochondrial-mediated apoptosis [6,7]. Lipids, including phospholipids, cholesterol, and ceramide, are synthesized in the MAM and transferred to the OMM [1,8,9]. In addition, the MAM is enriched in internal lipid rafts [10], which can serve to connect extrinsic and intrinsic apoptotic pathways [11]. Finally, mitochondrial antiviral responses have also been recently linked to the MAM [7,12,13].

The viral mitochondria-localized inhibitor of apoptosis (vMIA), encoded by the human cytomegalovirus (HCMV) UL37 exon 1 (UL37x1) immediate early gene, inhibits mitochondrial- mediated programmed cell death and increases viral progeny production during permissive infection [14,15,16,17,18,19]. vMIA traffics sequentially from the ER to mitochondria and is present at ER‑mitochondria contact sites known as MAM [20,21,22,23,24]. At the ER, vMIA causes ER Ca^2+^ efflux [15]. It associates with MAM lipid rafts in close proximity to sigma 1 receptor (Sig-1R) [25], which is a chaperone affecting Ca^2+^ efflux from the ER. Additionally, vMIA recruits Bax to MAM lipid rafts and induces Bax proteasome-mediated degradation, thereby augmenting vMIA’s antiapoptotic activity [26,27]. Because of its sequential trafficking [20,23], vMIA can relocalize a cellular defense protein, viperin, from the ER to mitochondria where viperin assumes a new role of a major effector to induce lipogenesis metabolism during HCMV infection [28,29]. At mitochondria, vMIA blocks Bax-mediated permeabilization of the OMM [17,18,30,31], reduces ATP synthesis [28,29,32], causes mitochondrial fragmentation [12,30,33,34], and controls HtrA2/Omi-induced cell death through very late times of HCMV infection [35].

vMIA is N-terminally anchored to ER and mitochondrial membranes by an uncleaved hydrophobic leader and its downstream C-terminal sequences are cytosolic [20]. This topology was confirmed by vMIA’s sensitivity to protease digestion in ER and mitochondrial fractions [22]. Immune electron microscopy (EM) of stably transfected HeLa cells has localized vMIA-myc using anti-myc antibody primarily at the OMM [16]. However, fixation, embedding and staining of specimens for EM severely compromise membrane morphology. Moreover, immune EM limits identifying the distribution of the protein populations such that only a fraction of the molecules in a given cellular organelle can be detected, thereby offering limited information about spatial distribution of the targeted molecule. Thus, the exact pattern of vMIA distribution and functional organization along the mitochondria was not detected by immune EM analysis. This EM analysis aside, vMIA imaging has primarily utilized conventional confocal microscopy [15,16,17,18,20,23,24,30,31,32,35,40]. Using multicolor confocal microscopy, we have previously found that enhanced green fluorescent protein (EGFP) tagged vMIA partially co-localizes with MAM, lipid raft, and mitochondrial markers [20,21,23]. Further, vMIA has been co-localized with mitochondrial markers from the OMM and matrix [17,18,20,21,23,24,25,32].

A major challenge in precisely defining vMIA’s localization in the ER, MAM and sub-mitochondrial compartments by confocal microscopy results from the close proximities of the ER and OMM membranes (10–25 nm) and of the OMM and inner mitochondrial membrane (IMM) at the MAM, which are below its diffraction limit. While confocal microscopy can theoretically produce a resolution down to ~200 nm, this resolution of visible light is seldom achieved in practice due to numerous optical aberrations associated with biological specimens as well as noise associated with the detected fluorescence. Some of this can be corrected by deconvolution of confocal images [41]. Mitochondria typically have a diameter of 200–500 nm [38]. Thus, it is not possible to determine vMIA distribution within sub-mitochondrial compartments using conventional confocal microscopy. For this, we turned to superresolution microscopy, which allows imaging beyond the limitations imposed by diffraction, to improve insight into vMIA’s distribution in sub-mitochondrial compartments.

Superresolution microscopy overcomes the physical limit imposed by diffraction. Multiple approaches have been developed to resolve fluorescent signals below diffraction limit and these include structured illumination (e.g., structured illumination microscopy, SIM; multifocal SIM, MSIM), reduction of point spread function by grounding emissions outside of the excitation center (e.g., stimulated emission depletion, STED; gated STED, GSTED) or activation of single fluorophores (e.g., photoactivated localization microscopy, PALM; stochastic optical reconstruction microscopy, STORM). Each of these approaches has its own strengths and weaknesses. In this study, we examined the localization of vMIA by deconvolved confocal microscopy and three superresolution microscopy techniques namely MSIM, single-color GSTED, and PALM.

MSIM uses sparse 2D excitation patterns [42] moved in sequential steps to fully illuminate the specimen. Images are collected at each step and used in post processing to derive a superresolution image. Superresolution is achieved by first defining the precise location of the illumination spots. Once the focal spots in each of the images are defined, these are digitally pinholed followed by scaling the spots by a factor of 0.5 and then summing these images over all positions. The pinholed, scaled, and summed images are then subjected to Richardson-Lucy deconvolution to gain ~2-fold improvement in resolution. Although MSIM sacrifices speed compared to confocal microscopy, it maintains the optical sectioning and was shown to provide resolution-doubling characteristics of SIM to ~140 nm [42].

Further improvement in resolution over MSIM is obtained using STED microscopy, where a 592 nm wavelength doughnut shaped beam is used to drive the fluorochromes in the doughnut to ground state by stimulated emission resulting in <50 nm resolution [43,44]. Time gating of the short fluorescent lifetimes caused by stimulated emission results in the GSTED approach further improves the spatial resolution. STED microscopy has previously shown that the mitochondrial inner membrane organizing system (MINOS) forms clusters within mitochondria of primary human fibroblasts [45] while two color STED found VDAC type 3 and hexokinase I clusters on the OMM of human osteosarcoma cells [46]. Similarly, cytochrome c oxidase subunit 2 and VDAC1 were found in clusters in purified mitochondria from murine heart [47].

Pointillistic imaging based superresolution microscopy approach, PALM offers the highest resolution microscopy (~25 nm) used in these studies. PALM uses photoactivatable fluorescent proteins and precise localization of single molecules to overcome diffraction limitations. PALM is based on high density, single molecule localization in which single molecule signals are fitted with 2D Gaussian functions to provide a more precise estimate of the molecule’s location. PALM and several related techniques use photoactivatable, photoswitchable, or photoconvertible fluorescent proteins [48], which initially have little fluorescence or their fluorescence can be turned “off” in the spectral region under detection before they are actively turned “on” during imaging. Conservatively setting the precision cutoff at ~50 nm can often produce images resolved at that value which is ~3-fold improvement over the MSIM images and comparable to the GSTED images. Similar to MSIM, PALM provides the ability to image multiple colors and better resolution than confocal, but slower imaging speed.

## 2. Results and Discussion

### 2.1. Conventional Confocal Imaging of vMIA Localization with Mitochondrial Markers

**Figure 2:**
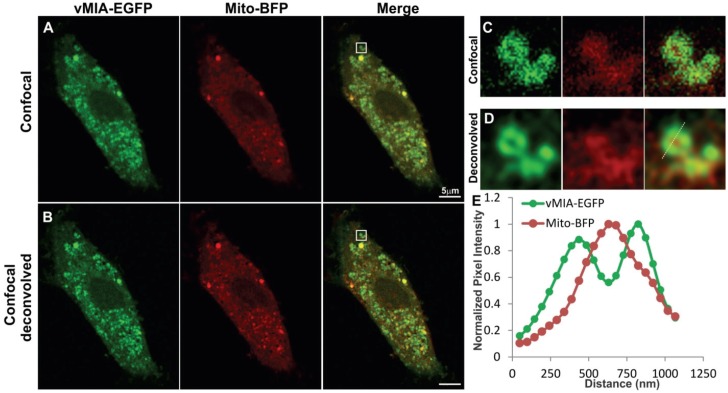
Monitoring mitochondrial localization of vMIA by confocal microscopy. Primary human foreskin fibroblasts (HFFs) lipofected with vectors expressing vMIA-EGFP and Mito-BFP were fixed with 4% paraformaldehyde (PFA) at 22 hours after transfection as described in the methods. (**A**) Images show a single optical plane for a cell expressing vMIA-EGFP (green) and Mito-BFP (pseudocolored red) imaged using confocal microscopy and (**B**) the same image plane following deconvolution of the entire Z-stack. (**C**,**D**) The boxed region of interest is enlarged on the right. (**E**) Intensity profile of vMIA-EGFP (green) and Mito‑BFP (red) emissions along the pixels marked by the dotted line on the deconvolved image are shown by the plot. For higher resolution images, see Supplemental Figure S1.

We first used confocal microscopy to monitor mitochondrial distribution of vMIA in HCMV permissive cells, human foreskin fibroblasts (HFFs) expressing vMIA-EGFP, which traffics indistinguishably from untagged vMIA [15,21,23,26]. We labeled the mitochondrial matrix by using *S. cerevisiae* mitochondrial Cox 4 leader fused to Tag blue fluorescent protein (Mito-BFP) [37] (Figure 2). As we previously found [20,21,23,24], vMIA-EGFP fluorescence substantially co‑localized with a matrix marker, Mito-BFP [37] (Figure 2A). The zoom of one of the larger mitochondrion (Figure 2C) shows an example of the level of detail available when imaging these markers with confocal microscopy. With the spatial resolution we obtained for this image (FWHM for vMIA-EGFP = 298 nm), vMIA-EGFP distribution was only marginally distinguishable from Mito‑BFP. With the image acquired 3-times below the Nyquist limit, deconvolution of the confocal Z‑stack images improved the spatial resolution (FWHM for vMIA-EGFP = 182 nm) (Figure 2B). This improved resolution allowed us to distinguish the presence of vMIA at the rim of the mitochondria, away from the matrix marker Mito-BFP (Figure 2D). This is quantified by the intensity profile for the dotted line marked along the deconvolved image of the mitochondrion (Figure 2E). This imaging also demonstrated that vMIA is not uniformly distributed along the mitochondrial periphery, hinting at the possibility of clustering of vMIA on the mitochondrial surface (Figure 2D); however, the clusters of molecules were not clearly resolvable even after deconvolution of the confocal microscopy images. In summary, confocal microscopy showed the presence of vMIA at the periphery of mitochondria and partially resolved it from the matrix marker.

**Figure 3:**
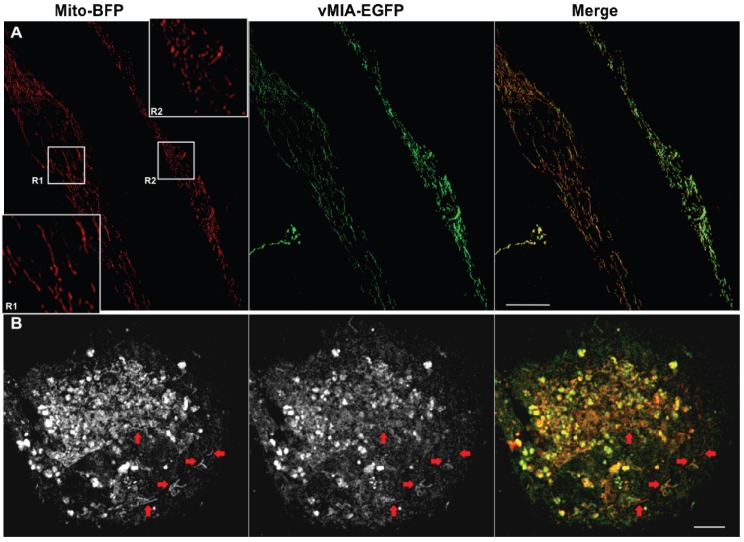
Mitochondrial fragmentation by vMIA exhibits a threshold effect. Primary HFFs transfected with vectors to express vMIA-EGFP and Mito-BFP were fixed with 4% PFA at 22 hours after transfection as described in the methods and visualized by confocal deconvolution microscopy. (**A**) Maximal intensity projection of a 3-D image showing a pair of cells expressing differing levels of vMIA-EGFP (green), but comparable level of Mito-BFP (pseudocolored red). Insets showing the zoom of regions R1 and R2 highlights the change in mitochondrial morphology (as shown by Mito-BFP) in cells expressing low level (R1) or high level (R2) of vMIA-GFP (**B**) Maximal intensity 3-D projection of another cell expressing Mito-BFP (pseudocolored red in the merge) and showing varying levels of vMIA-EGFP (green in the merge) on the individual mitochondria. While most of the mitochondria in this cell have lost their tubular appearance, red arrows point to the individual mitochondrion that show low or no vMIA-GFP expression have remained tubular. Scale bars represent 5 µm. For higher resolution images, see Supplemental Figure S2.

In these studies, we detected altered mitochondrial morphology in HFFs expressing vMIA consistent with previous literature [30,33,34,49]. To determine if the altered mitochondrial morphology correlated with vMIA levels, we examined mitochondria morphology in cells expressing vMIA (Figure 3). We found that mitochondria (red) not expressing vMIA or expressing low levels of vMIA (R1) maintained tubular morphology (Figure 3A). Conversely, mitochondria expressing higher levels of vMIA (R2) showed fragmented, vesicular morphology. Multiple tubular mitochondria (blue arrows) not expressing or expressing low levels of vMIA were also observed in another cell (Figure 3B). These results suggest that threshold levels of vMIA are required for mitochondrial fragmentation and vesiculation.

**Figure 4:**
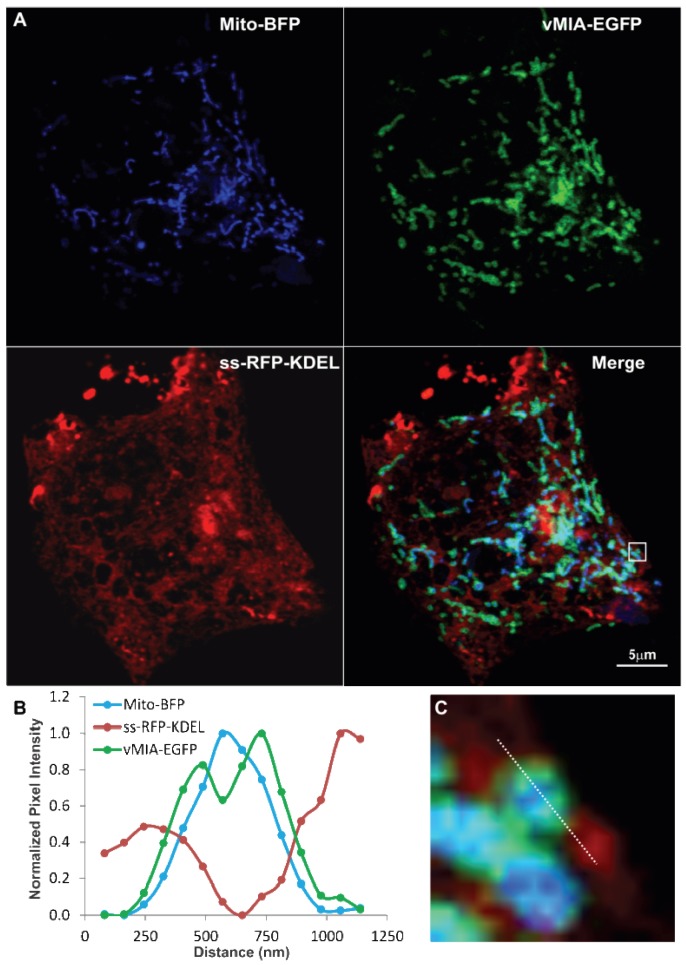
Confocal microscopy imaging of ER-mitochondria interface. HFFs were lipofected with vectors expressing vMIA-EGFP (OMM), ss-RFP-KDEL (ER) and Mito*‑*BFP (matrix) and fixed with 4% PFA at 25 hours after transfection as described below and previously published [23]. (**A**) Cells expressing vMIA-EGFP (green), ss-RFP-KDEL (red) and Mito-BFP (blue) were imaged using confocal microscopy. (**C**) The boxed region of interest from the merged image is enlarged. (**B**) Line scans of vMIA*‑*EGFP (green), ss-RFP-KDEL (red) and Mito-BFP (blue) emissions are shown. For higher resolution images, see Supplemental Figure S3.

With the ability of the deconvolved confocal image to resolve vMIA distribution from the mitochondrial matrix marker (Mito-BFP), we next examined the distribution of these markers with respect to that of the ER (ss-RFP-KDEL), which has the bovine preprolactin signal sequence (ss) fused to monomeric red fluorescent protein (RFP) with the KDEL ER retention [36] sequence (graciously provided by Dr. J. Lippincott-Schwartz) (Figure 4A). HFFs expressing the three fluorophore-tagged proteins showed that vMIA partially colocalized with ER and mitochondrial markers as we previously found [20,21,23,24,26,50]. Moreover, the intensity profile (Figure 4B) of the pixels along the line shown in the zoomed boxed region of interest (Figure 4C) showed that vMIA-EGFP is located at the interface of Mito-BFP and ss-RFP-KDEL. Although there is detection of the interfaces between the ER and mitochondria, there is substantial overlap of the mitochondrial OMM (green) and matrix (blue) sub-compartments, which limits the ability of confocal microscopy to compellingly resolve the sub‑mitochondrial distribution of vMIA.

### 2.2. Single Color GSTED Imaging of vMIA Localization

**Figure 5:**
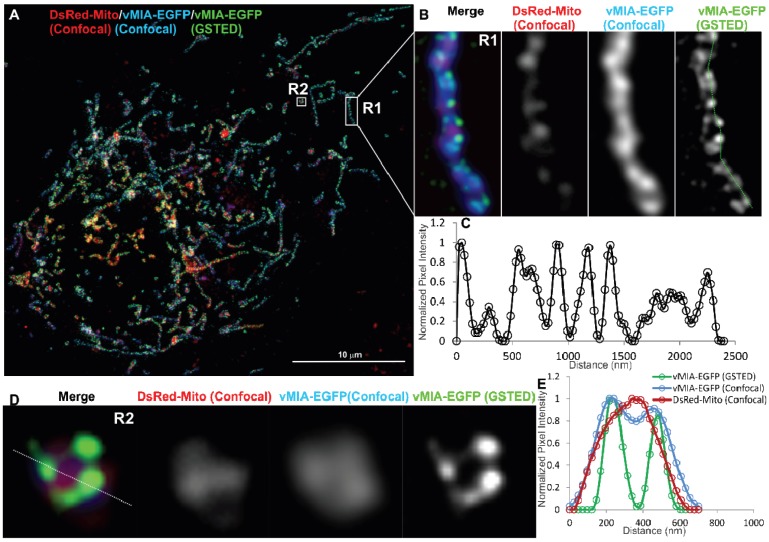
Gated stimulated emission depletion (GSTED) microscopy of vMIA-EGFP in human fibroblasts. (**A**) HFFs were lipofected with vectors expressing vMIA-EGFP and DsRed-Mito. At 24 hours post transfection, cells were methanol fixed as described as below and elsewhere [23] and imaged using GSTED (vMIA-EGFP) and confocal microscopy (DsRed-Mito, vMIA‑EGFP) followed by deconvolution of both the images. (**B**) Zoomed, merged image of a tubular mitochondrion in the boxed region (R1) is shown. This includes DsRed-Mito confocal (red), vMIA-EGFP confocal (blue) and vMIA-EGFP GSTED (green). Each channel is also presented individually. (**C**) Intensity profile of the pixels marked by the dotted line on the GSTED panel demonstrates the clustered distribution of vMIA along the entire length of the OMM of this mitochondrion. (**D**) The zoomed, merged image of a mitochondrion in the boxed region (R2) is shown. (**E**) The normalized intensity profile along the line shown on the R2 image, which demonstrates the significant improvement in visualizing the vMIA distribution along OMM by GSTED as compared to confocal imaging and its improved resolution of its localization away from the matrix and at the OMM. For higher resolution images, see Supplemental Figure S4.

To image vMIA beyond the diffraction limit imposed by the visible light, we used GSTED, an earlier variant, STED, has been used to study clustered distribution of several mitochondrial proteins at the OMM and IMM by immune localization of intact or isolated mitochondria [45,46,47]. As use of antibody affects precision of localization due to increased distance added on by the presence of primary and fluorescently tagged secondary antibodies and imaging isolated mitochondria provides localization outside the biologically relevant subcellular context, we undertook *in situ* imaging of vMIA-EGFP in permissive HFFs (Figure 5A). Following deconvolution of the GSTED image, we detected vMIA-EGFP (green) in the periphery of a tubular mitochondrion (Figure 5B), which is consistent with the above suggestion of OMM localization by the confocal microscopy and of biochemical literature [16,20,22]. Intensity profile of pixels marked by the line shown in the zoomed GSTED image of the region R2 confirmed that vMIA is localized at the periphery of mitochondria, distinguishable from DsRed-Mito, used as a matrix marker and imaged by confocal microscopy (Figure 5D,E). GSTED showed improved resolution of vMIA (FWHM = 75 nm) OMM location compared to confocal imaging of vMIA (Figure 5B). This increase in spatial resolution also offered conclusive evidence to support clustered distribution of vMIA along the OMM (Figure 5C). Thus, superresolution GSTED resolved the presence of vMIA at the OMM and its clustered distribution in mitochondria in permissive HFFs. It also showed that vMIA exists as <100 nm clusters at the OMM irrespective of if the mitochondrion is tubular (Figure 5B) or fragmented (Figure 5D).

**Figure 6:**
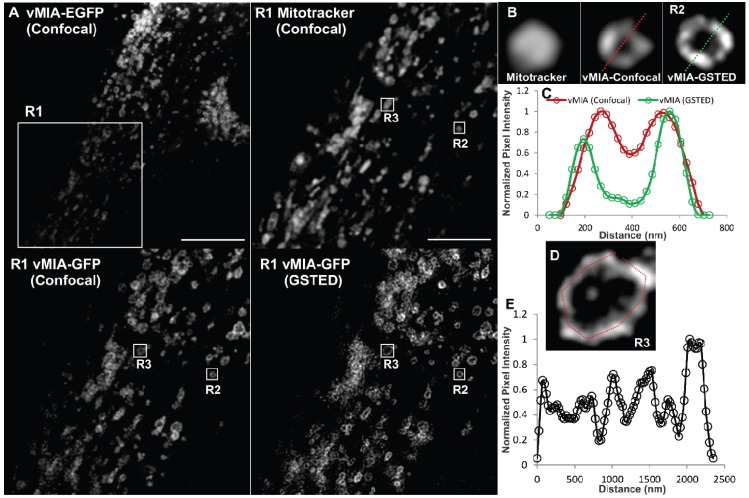
GSTED microscopy of vMIA-EGFP in human astrocytoma cells. U373-Tet-ON cells expressing the tetracycline controlled transactivator (tTA) [51] were lipofected with the tetracycline responsive element (TRE-Tight) promoter driving expression of vMIA-EGFP. At 24 hours post transfection, cells were treated with doxycycline (Dox) for 60 minutes, labeled with MitoTracker Red (0.5 µM) and imaged live using Confocal (vMIA-GFP and MitoTracker Red) and GSTED (vMIA-GFP). (**A**) An optical slice showing deconvolved confocal image of a cell expressing vMIA-EGFP and a zoom of the region corresponding to the boxed region (R1) are shown in the left panel. Right panel presents the zoom of the R1 region showing the deconvolved GSTED (bottom) and deconvolved confocal MitoTracker (top) channels. (**B**) Zoomed confocal images of the mitochondrion in the region R2 showing the various channels acquired*—*Confocal images of MitoTracker Red and vMIA-EGFP as well as GSTED image of vMIA-EGFP. (**C**) Normalized intensity profile along the dotted line shown on the confocal and GSTED images of R2 demonstrate the improved resolution of vMIA localization by GSTED imaging as compared to confocal imaging. (**D**) Zoom of region R3 showing GSTED image of vMIA (**E**) Intensity profile of the pixels marked by the red line in panel (**D**). For higher resolution images, see Supplemental Figure S5.

As clustering of vMIA along the OMM is an unprecedented phenotype for a viral protein, we performed GSTED imaging of vMIA-EGFP in another permissive (human astrocytoma) cell line, U373-Tet-ON cells [51] (Figure 6A). GSTED imaging detected vMIA-EGFP (green) in U373-Tet-ON cells also showed that similar to HFFs, vMIA is present at the rim of the mitochondria (Figure 6B). This rim-like distribution of vMIA-EGFP was confirmed by the intensity profile of pixels marked by the line shown in the zoomed region (R2, Figure 6B). GSTED showed improved resolution of vMIA location (<50 nm) compared to confocal imaging (200 nm) (Figure 6C). Similar to our findings in HFFs (Figure 5), vMIA was present in clusters on the mitochondria in transfected U373-Tet-ON cells (Figure 6D,E). However, mitochondria of the U373-Tet-ON cells expressing vMIA are considerably less tubular (Figure 6) than mitochondria from transfected HFFs expressing vMIA (Figure 5). These differences in mitochondrial morphology likely represent cell-type specific differences in the effects of vMIA, which is known to alter mitochondrial morphology [30,32,33,52].

### 2.3. MSIM Imaging of vMIA and Mitochondrial Marker

**Figure 7:**
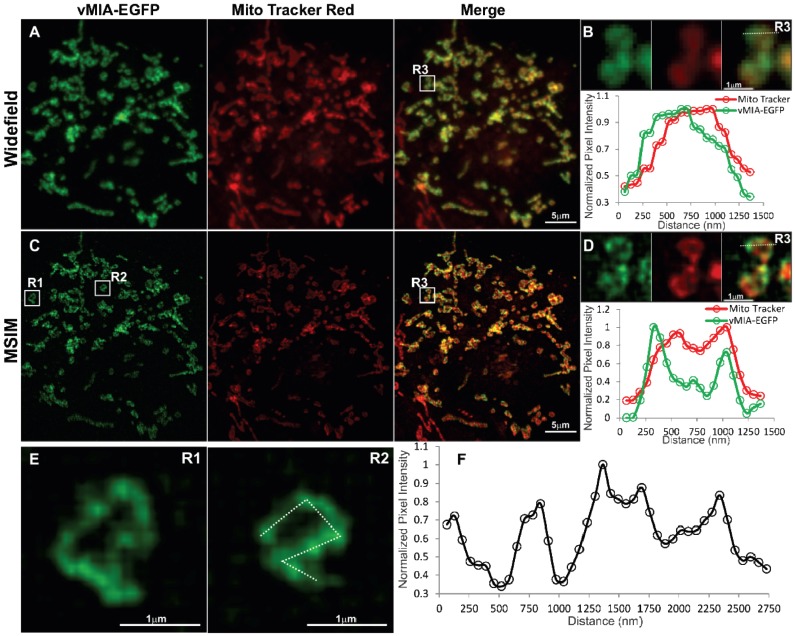
Imaging vMIA localization using widefield microscopy and MSIM. HFFs were transfected with vectors expressing TRE-tight promoter-vMIA-EGFP (green) and its tetracycline controlled transactivator (tTA) [53]. Transfected cells were Dox-treated (0.2 µg/mL) for one hour, MitoTracker Red treated (0.5 µM for 5 min, red) and methanol fixed as described [23]. 25 slices (0.2 µm step size) were collected using 488 nm and 561 nm excitation lasers. For each channel, 256 multifocal excited images were collected as the excitation array was stepped ~1 pixel in a 16 × 16 grid pattern. (**A**) Summation of the images produces the widefield fluorescence image, which shows high degree of co‑localization between vMIA-EGFP and MitoTracker Red. (**B**) Zoomed images of mitochondria in the region marked R3 are shown and the plot below shows the intensity profile through the region marked by dotted line. (**C**) The MSIM image is shown. (**D**) The zoomed images of region R3 and the line scan for the region marked by dotted line show the improved ability to resolve the vMIA-EGFP and MitoTracker Red staining through the use of MSIM. (**E**) MSIM images of two mitochondria (R1 and R2) are shown. (**F**) Intensity profile through the perimeter of mitochondrion in R2 marked by dotted line is shown in the plot. This indicates non-uniform distribution of vMIA-GFP along the mitochondrial membrane. For higher resolution images, see Supplemental Figure S6.

With a restricted set of compatible fluorophores that can be used for multicolor superresolution imaging by GSTED, for simultaneous superresolution imaging of vMIA-EGFP in context of organelle markers we made use of an alternative superresolution imaging approach—MSIM. We performed MSIM imaging of doxycycline (Dox) treated HFFs dually transfected with the tetracycline transactivator [53] and the TRE-Tight promoter driving vMIA-EGFP (Figure 7). We observed that vMIA localized distinctly from the mitochondrial marker we used here—MitoTracker Red, which appears to localize to the IMS [38]. As expected, widefield imaging of vMIA-EGFP and MitoTracker Red showed predominant colocalization of the two fluorophores (Figure 7A). With the observed spatial resolution of 302 nm for this image there was little resolution of the vMIA and MitoTracker labeling (Figure 7B). The overlap of the signals was documented by the intensity profile of each fluorophore along the pixels marked by the dotted line shown in the zoom of the region R3 (Figure 7B). MSIM images of the two fluorophores for this cell showed improved resolution of vMIA‑EGFP and MitoTracker Red (Figure 7C). Intensity profile of MSIM image of each fluorophore along the pixels marked by the dotted line shown in the zoom of the region R3 showed partial separation of the vMIA-EGFP fluorescence peaks from those of the MitoTracker fluorescence peaks such that the bimodal vMIA peaks are farther away to the outside of the bimodal MitoTracker peaks (FWHM = 120nm) (Figure 7D). This distribution of the two markers is in agreement with the vMIA localization to the OMM, surrounding the IMS localized MitoTracker Red. Furthermore, MSIM imaging in individual mitochondrion (R1 and R2 in Figure 7E) confirmed our previous observations of vMIA localization in clusters at the OMM. The presence of vMIA clusters was verified by intensity profile of MSIM image of each fluorophore along the pixels marked by the dotted line drawn along the rim of a mitochondrion in R2 (Figure 7F). These vMIA clusters were obscured by diffraction in widefield imaging (Figure 7B) and confocal microscopy (Figure 2, Figure 3 and Figure 4) but detected by superresolution GSTED imaging (Figure 5 and Figure 6). Together, these results show that MSIM partially resolves vMIA location at mitochondria periphery, away from IMS, and in clusters of diffraction limited size. The ER and OMM make contacts at the MAM. In addition, using superresolution microscopy multiple mitochondrial proteins including the OMM VDAC1, VDAC3 and Tom 20 proteins have been shown to exist in clusters as functional mitochondrial complexes [46,47,54]. vMIA clustering could represent the contact sites between the ER and OMM or could indicate the vMIA association with functional complexes at the OMM. Use of above approaches allowed us to narrow down the size of vMIA clusters to be in the range of 100 nm, but this is close to the resolution of imaging modalities used.

### 2.4. PALM Imaging of PA-mCherry-vMIA

**Figure 8:**
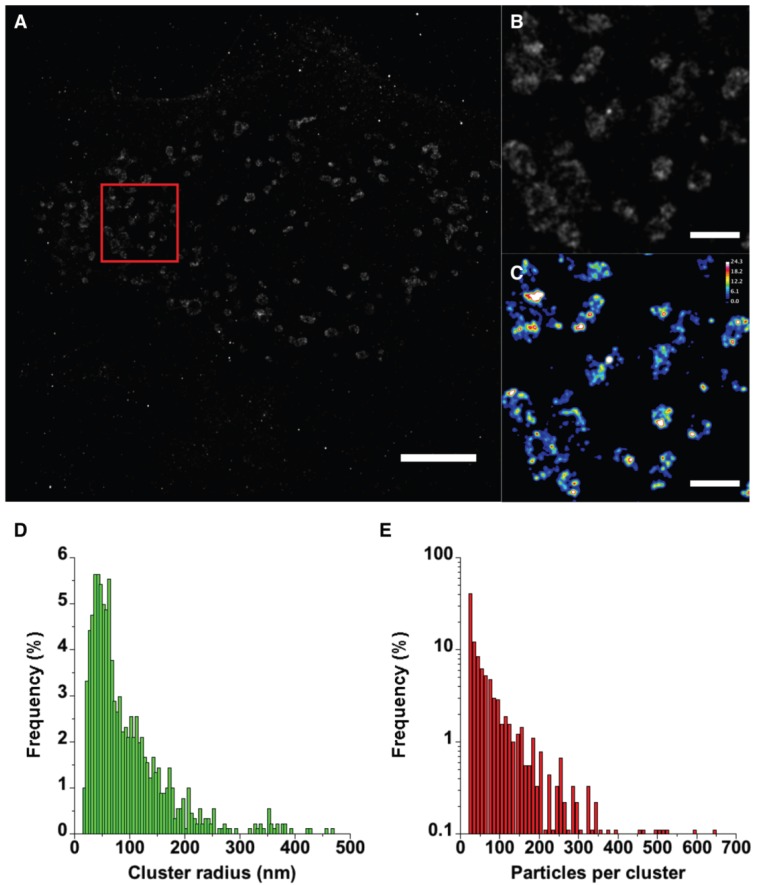
PALM imaging of vMIA-PAmCherry1. HFFs were transfected with vMIA-PAmCherry and imaged by PALM. (**A**) The image shows 301666 molecules that were localized to ≤25 nm precision out of 515207. Molecules are plotted as two-dimensional Gaussian distributions with 25 nm standard deviations. (**B**) The image shows a small region in (**A**) indicated by the red square. The molecule positions in a binary image were used in pair correlation analysis and the maximum g(r) derived from using each molecule as a reference point was plotted as the pixel values. (**C**) The image shows the same region in the red square color-coded to display the varying degrees of molecule densities. The Look Up Table (LUT) and calibration bar are included in the upper right corner. The distributions of cluster sizes (**D**) and the number of particles per cluster (**E**) were determined from the pair correlation analysis results. The maximum cluster radius was limited to 500 nm and the minimum number of particles per cluster was limited to 20. Please note that the Y axis in (E) is a log scale to better indicate the lower percentages at the higher particle numbers. The scale bar in (**A**) is 5 μm and in (**B**, **C**) is 1 μm. High resolution images of the entire field of view are available in the SOM (see Supplemental Figures S7, S10 and S11).

To visualize vMIA clusters by yet another independent superresolution approach and obtain a better estimate of the size of vMIA clusters, we made use of the pointillistic imaging superresolution imaging approach PALM. Here, we expressed vMIA tagged with PAmCherry, a photoactivatable red fluorescent protein [55], in HFFs and visualized them using 2D PALM imaging (Figure 8). Most of the molecules in this image localized with precision better than 25nm, but here we have conservatively rendered all molecules which have been localized to <25 nm precision as 2D Gaussian distributions with 25 nm sigmas. Similar to our MSIM and GSTED results above, qualitative observations suggest a non-uniform distribution of vMIA-PAmCherry on the periphery of the mitochondria when rendered in this manner. PALM imaging also affords a second, rather straightforward analysis of molecule distributions, which is relevant to this work, cluster analysis. Several methods are developed for this sort of analysis, but we opted for pair correlation analysis in quantitatively determining which regions of each mitochondrion displayed increased vMIA protein densities.

In these analyses, the function g(r) is determined by comparing the protein density in a local “shell” region around each position with the average density of all molecules of interest (see Supplemental Figure S8). Simulated images containing various levels of clustering are shown in Supplemental Figure S9A–D. For these, the factor g is plotted as a function of the radius and for images containing no clusters, this remains close to 1 as the radius decreases toward zero (Supplemental Figure S9E). However, in images with clusters, the g(r) deviates from 1 at approximately the diameter of the clusters (Supplemental Figure S9E). If the cluster radii increase, the g(r) deviates at a higher value of r. If the molecule density in the cluster increases, the maximum g(r) increases. Thus, the maximum g(r) can reflect the relative amplitude of the molecule density surrounding each reference particle. This is important for our simple analysis in which we compare the local protein density with the whole image density. With these criteria, the entire mitochondrion could be considered a cluster. In our analyses, we used the average density of all molecules in the image localized to <25 nm precision. We plotted the g(r) maximum value obtained for each reference molecule in a new image at the appropriate location (Supplemental Figure S10). Indeed, we find that most of the molecules localized to the mitochondria show g(r) values greater than 1, but we also note that regions resembling the clusters observed with MSIM and GSTED have much higher g(r) values than surrounding peripheral regions (Figure 8). Using the results from pair correlation analysis, the cluster sizes (Figure 8D) and the number of particles per cluster (Figure 8E) were determined. Cluster radii show a broad distribution with a mean radius of approximately 95 nm and suggest that a majority are <100 nm. The number of particles per cluster is also broadly distributed and indicates that a majority of the clusters (approximately 60%) contain 50 particles or less.

## 3. Experimental Section

### 3.1. Cell Culture and Lipofection

HFFs were cultured in Dulbecco’s Modified Eagle’s medium containing 10% fetal calf serum (FCS, Hyclone, Logan, UT), 100 U/mL of penicillin, 100 μg/mL of streptomycin, 2 mM L-Glutamine (Life Technologies, Grand Island, NY) as previously described [56]. U373-Tet-ON cells were also cultured in the 10% FCS, 100 U/mL of penicillin, 100 μg/mL of streptomycin, 2 mM L-Glutamine but maintained under selective pressure using 0.6 mg/mL Geneticin (Life Technologies).

Cells were seeded at 20%–50% confluency on sterile 18 mm (for confocal or GSTED) or 25 mm (for PALM and MSIM) cover slips in six-well plates (9.72 cm^2^ per well). Twenty-four hours later cells were transiently transfected using Lipofectamine 2000 (Life Technologies) suspended in Opti-MEM (Life Technologies), according to the manufacturer’s protocols. DNA (µg):lipid (µL) ratios for transfection were at 1:1.77, with approximately 0.5 µg total DNA used per cm^2^ of available plating surface area. Cells were harvested 22-25 hours after transfection by fixation with methanol or 4% PFA in PBS and mounted with Prolong Gold Antifade. GSTED imaging was performed on live U373-Tet-ON cells treated with Dox (0.5 µM).

### 3.2. Construction of pTRE-Tight Promoter-vMIA-EGFP

The vMIA/UL37x1 protein-EGFP open reading was isolated from p1242 [15] by restriction enzyme digestion with EcoRI and NotI. The fragment was ligated into EcoRI/NotI digested TRE‑Tight vector (Clontech, Mountain View, CA, USA) and the ligation product was transformed into competent *E. coli* (strain DH5 alpha).

### 3.3. Confocal Microscopy

The confocal images were acquired using the Olympus FV1000 confocal microscope. An UPlanSApo100x/1.40NA oil objective was used to obtain an oversampled 1024 × 1024 image with 49 nm pixel and a z-stack with a step size of 120 nm. The Mito-BFP and the vMIA-EGFP were sequentially excited using a 405nm diode laser and a 488nm Argon laser, respectively, and collected between 425 nm–475 nm and 500 nm–545 nm in the spectral detectors. In the triple labeled cells, ss‑RFP-KDEL was also sequentially collected using 575 nm–675 nm filter after excitation with a 559 nm diode laser.

### 3.4. GSTED Microscopy

The GSTED images were acquired using a Leica TCS SP5 gated STED (GSTED) microscope that was equipped with a super continuum white light laser (WLL) and hybrid detectors adapted for time gated imaging which allow elimination of low spatial frequency from the final super resolved image [57]. The 592 nm depletion laser delivers 0.3W at the focal plane. A HCX PL APO 100× 1.40 NA oil objective was used to obtain a 1600 × 1600 image with a 24.2 nm pixel. The DsRed-Mito confocal image was acquired using the 560 nm excitation and collected between 569 nm–665 nm. A sequential GSTED image of UL37-EGFP was obtained using a 488 nm excitation, 592 nm depletion, time gating from 1–8 ns, and collected between 500 nm–544 nm. Using the tunability of the white light laser and prism based spectral emission. “Lambda lambda” scans were acquired for an excitation range of 470 nm–590 nm and an emission range of 485 nm–605 nm. Both excitation and emission scans were taken with 10 nm wavelength steps between measurements and was used to confirm the excitation and emission properties of the EGFP fluorophore imaged on this microscope in the GSTED or confocal mode.

### 3.5. Deconvolution Analysis

Blurring due to out of focus signal and Poisson noise in the confocal and GSTED images was removed by carrying out deconvolution using the Huygens Essential software supplied by Scientific Volume Imaging B.V. (Hilversum, The Netherlands). For confocal images, deconvolution was done using 3D images acquired as described above. For GSTED images, 2D deconvolution was done on the using images acquired as discussed below. For generating pixel intensity plots images were imported and analyzed in the Metamorph Premier (7.7.0) software supplied by Molecular Devices, LLC (Sunnyvale, CA, USA).

### 3.6. MSIM

The MSIM microscope used for these experiments is a homebuilt machine using an Olympus IX-71 widefield microscope as previously described [58] and with modifications detailed below. A 100 mW 405 nm Cube laser (Coherent, Inc., Santa Clara, CA, USA), 50 mW 488 laser (Oxxius, Lannion, France, LBX-488-50-CIR-PP), 50 mW 561 Sapphire laser (Coherent, Inc.), and 100 mW 640 nm Cube laser (Coherent, Inc.) served as illumination sources for blue, green, red, and far-red fluorophores, respectively. The light from each laser was filtered by placing a 405/10 nm BrightLine single-band bandpass filter (Semrock Inc., Rochester, NY, USA, FF01‑405-10/25) at the aperture of the 405 laser, a 488/6 nm BrightLine single-band bandpass filter (Semrock Inc., FF01-488-6/25) at the aperture of the 488 laser, a 561/4 nm BrightLine single-band bandpass filter (Semrock Inc., FF01-561-6/25) at the aperture of the 561 laser, and a 640/8 nm MaxDiode laser clean-up filter (Semrock Inc., LL01-640-8/12.5) at the aperture of the 640 laser. The laser beams were collimated or expanded and collimated with insertion of pairs of lenses after the clean-up filters. The 405 laser has a 2× telescope consisting of a 50 mm focal length lens (Thorlabs Inc., Newton, NJ, USA, LA1131-A) and a 100 mm focal length lens (Thorlabs Inc., LA1509-A). The 488 laser has a 1× telescope consisting of a pair of 100 mm focal length lenses (Thorlabs Inc., LA1509-A). The 561 laser has a 3× telescope consisting of a 50 mm focal length lens (Thorlabs Inc., LA1131-A) and a 150 mm focal length lens (Thorlabs Inc., LA1433-A). The 640 laser has a 2× telescope consisting of a 50 mm focal length lens (Thorlabs Inc., LA1131-A) and a 100 mm focal length lens (Thorlabs Inc., AC254-100-A). Mechanical shutters along with acousto-optic tunable filter (AA Opto-electronic Inc., Orsay, France, AOTFnC-400.650) controlled by Micro-Manager [59] allowed for laser wavelength selection, laser power tuning and laser shuttering. The laser beams were expanded 5× using a 40 mm focal length lens (Thorlabs Inc., AC254-40-A) and a 200 mm focal length lens (Thorlabs Inc., AC254-200-A). Collected fluorescence was filtered through a 480/40 bandpass filter (Chroma Technology Corp, Bellows Falls, VT, USA D480/40m) for 405 nm excitation, a 525/45 bandpass filter (Semrock, FF01-525/45-25) for 488 nm excitation, a 600/37 bandpass filter (Semrock, FF01-600/37-25) for 561 nm excitation, and a EdgeBasic 635 longpass filter (Semrock, BLP01-635R-25) for 640 nm excitation. All other components in the excitation and emission paths, such as optical components, 2D galvanometer, z‑stage and camera, are the same as previously published [58].

### 3.7. MSIM Data Collection and Analysis

MSIM data were collected for each slice as previously published [58] except the galvanometer was stepped in a 16 × 16 grid to collect 256 frames for each 512 × 512 pixel wide field of view. Post-processing was performed as previously published [58] on freely available software [60]. Deconvolution was performed on the MPSS images using a program written in Python (freely available at [60] implementing Richardson–Lucy deconvolution [58,61,62,63].

### 3.8. PALM Data Analysis

Single molecule images were analyzed as previously described [64] using PeakSelector (a program written in IDL by Harald Hess and Gleb Shtengel, Howard Hughes Medical Institute, Janelia Farms, Ashburn, VA, USA). The molecule localization data were output as an Ascii file which was used by an ImageJ macro (available upon request) [65] to plot the molecule positions and precisions on images with 5 nm pixels.

Pair correlation analysis was performed on binary images displaying peak positions rendered as single 5 nm pixels. The g(r) function was determined for each particle by using the equation

g(r) = ρ(δr)/ρ(A_N_)

where each particle is treated as a reference particle. The particle density in a “shell” region of δr is determined and then divided by the particle density in a region containing all of the particles of interest, A_N_. In these analyses, the entire image was used as A_N_ (Supplemental Figure S8). The analysis was performed using an ImageJ macro (available upon request) run in FIJI [66]. Briefly, the particle density ρ in region A_N_, ρ(δr), was determined by dividing the total number of rendered molecules by the area of the image. The particle density ρ in region δr, ρ(δr), was determined by first counting the number of particles in areas r1 and r2 (Supplemental Figure S8). The number of particles in region δr was determined by subtracting the r1 count from the r2 count. The area of δr was determined using πr^2^ by subtracting the area the r1 region from the area of the r2 region. The ρ(δr) was calculated by dividing the δr count by the area of δr.

The g(r) as a function of the radius r1 for each peak was fit with an exponential equation

g(r) = A × exp(–(1/B) × r) + C

where A is the amplitude, B is the radius of the cluster, and C is the offset. Only peaks with g(r) fitted with a correlation coefficient R^2^ > 0.9 were rendered in the g(r) image. To display these data, the molecule peak position image was rendered by setting the pixel values for each particle to the maximum g(r) determined for that peak when using it as a reference in the analysis (Supplemental Figure S10). A color coded LUT with calibration is included for comparison of different regions of the cell. Additional processing on this image included a Gaussian blur with a 25 nm sigma to represent the uncertainty in molecule localization (Supplemental Figure S11). 

## 4. Conclusions

Because superresolution microscopy is an emerging technology, it has had very limited use in the study of viruses. To date, superresolution imaging has primarily focused on RNA viruses including imaging of rotavirus virions [67], influenza hemagglutinin clusters [68], HIV assembly [64,69,70] and HIV transfer [71]. More recently, single copy viral genomes including those of DNA viruses have been imaged using superresolution microscopy [72]. To our knowledge, our studies are the first superresolution imaging of any human herpesvirus protein, in particular of a HCMV vMIA. 

HCMV vMIA imaging by confocal/deconvolution microscopy indicated its OMM localization, which was confirmed by GSTED, MSIM, and PALM imaging. Additionally these imaging modalities all demonstrate that at the OMM vMIA is present in clusters, indicating nanoscale localization of HCMV vMIA at the mitochondrial periphery, away from IMS and matrix. While this clustering is also suggested by deconvolved images obtained by the confocal microscopy, diffraction limits the ability to visualize the distribution of MAM clusters. This indicates that the vMIA domains are generally spaced closer than the diffraction limit. This inference has been supported by the ability to resolve them by each of the superresolution imaging approaches used here. Use of PALM imaging allowed us to estimate the sizes of these clusters at approximately 95 nm, which is in agreement with the cluster sizes indicated by the GSTED and MSIM approaches. 

Intriguingly, clusters of cellular mitochondrial proteins have been observed using superresolution microscopy. STED microscopy found that MINOS, which maintains IMM morphology, forms clusters often in an ordered inner mitochondrial distribution [45]. Tom 20 and Tom22, components of the translocase of the outer mitochondrial membrane, are clustered at the OMM with densities that correlate with mitochondrial membrane potential [73]. VDAC1 and VDAC3 have also been found by superresolution imaging to be distributed in clusters in the OMM. Cytosolic hexokinase I which associates with VDAC3 is partly also localized in clusters with VDAC3 [46,47]. Our superresolution imaging suggests that vMIA may target and associate with clustered OMM proteins. Alternatively, vMIA traffics efficiently to the MAM [20,21,22,23,26,74]. vMIA clustering may represent sites of contacts between the ER and OMM, where vMIA could be transferred between the two organelles. The use of vMIA mutants and known cellular markers of MAM should allow us to distinguish these possibilities. 

Superresolution imaging has the clear potential to provide valuable insight into the nanoscale organization of viral machineries, which provide essential replicative processes. Case in point is the detection of non-uniform distribution of vMIA at the OMM. This distribution was not detectable by conventional microscopy because of its diffraction limited resolution. Further, this technology allows virologists to study how viruses alter cellular organelles to establish replication compartments and virus assembly. However, the superresolution approach to be used should be carefully selected. Our approach here involved three techniques with complementary advantages in nanoscale studies of vMIA localization. GSTED relies primarily on imaging of one fluorophore impacting on the ability to place the viral proteins in the *in situ* biological context of cellular organelles or structures. Secondary markers can be imaged by GSTED using the appropriately labeled secondary antibodies (e.g., Alexa 532). GSTED provides greater resolution (<50 nm) than MSIM. However, use of primary and secondary antibodies can adversely affect precision of protein localization at the nanoscale resolution. Nonetheless, because of its resolution, GSTED provided compelling evidence for vMIA clustering in mitochondria and localization at the OMM. Conversely, MSIM is lower resolution than GSTED microscopy. However, MSIM makes use of the same fluorophores used for conventional confocal microscopy. This enables imaging of viral proteins in the context of multiple fluorophore tagged proteins from different sub-cellular compartments. For our experiments, MSIM provided sufficient resolution to confirm the location and clustering of vMIA at the OMM.

PALM imaging provides the highest resolution used in these studies but imaging sufficient molecules to obtain meaningful information requires hours of acquisition and post-acquisition processing. This limits most PALM imaging to fixed specimens. Given the increasing palette of photoactivatable fluorophores and increasing availability of these powerful microscopy techniques much can be learned by their application to multiple viruses and their processes.

Here, we have used three different superresolution techniques with multiple markers to image the localization and clustering of vMIA. These all confirm the presence of the clusters on the outer membranes of mitochondria and are helping to define the cluster physical characteristics. In general, the clusters are found closer together than the limits of resolution by widefield and confocal microscopy. Examples of these include the plot profiles in Figure 5, Figure 6 and Figure 7 compared with the widefield or confocal counterparts. These results show that given time, the imaging techniques used here will help discern on a sub-mitochondria scale where are the components of MAMs located in relation to each other as well as other cellular players.

A second important finding here is that we may put a conservative upper limit on the diffraction-limited vMIA cluster size of approximately 150 nm. Since this comes from the data collected with three independent imaging techniques, it provides a more reasonable estimate than simply relying on a single technique.

Why is the size of importance? At these scales, small cluster sizes dictate the maximum number of molecules which can fit inside. Given the number of processes in which MAMs are known to play a role, this imposes limits on the number of molecules which can participate in signaling events. Thus, every MAM may not have all of the components to initiate all processes and this brings up another nagging question in the MAM field. Are the MAMs homogeneous or have differing compositions which imply different functions? Now that these structures can be readily imaged with optical techniques using multiple color markers, answers to this question will be far more straightforward to derive than with previous efforts. In summary, our studies show that superresolution imaging provides valuable insight into sub-diffraction resolution of viral protein location, particularly in the sub‑mitochondrial compartments, and into their clustered arrangement.

## Supplementary Files

Supplementary File 1 Supplementary Information (PDF, 3325 KB)
