# Supplementary material for: Superresolution Imaging of Human Cytomegalovirus vMIA Localization in Sub-Mitochondrial Compartments
Source: Viruses. 2014 Apr 9;6(4):1612–36. doi: 10.3390/v6041612 (PMC4014713; doi:10.3390/v6041612)

Supplemental Figure S1. Monitoring mitochondrial localization of vMIA by confocal microscopy. Primary HFFs lipofected with vectors expressing vMIA-EGFP and Mito-BFP were fixed with 4% paraformaldehyde (PFA) at 22 hours after transfection as described in the methods. (**A**) Images show a single optical plane for a cell expressing vMIA-EGFP (green) and Mito-BFP (pseudocolored red) imaged using confocal microscopy and (**B**) the same image plane following deconvolution of the entire Z-stack. (**C**, **D**) The boxed region of interest is enlarged on the right. (**E**) Intensity profile of vMIA-EGFP (green) and Mito-BFP (red) emissions along the pixels marked by the dotted line on the deconvolved image are shown by the plot.

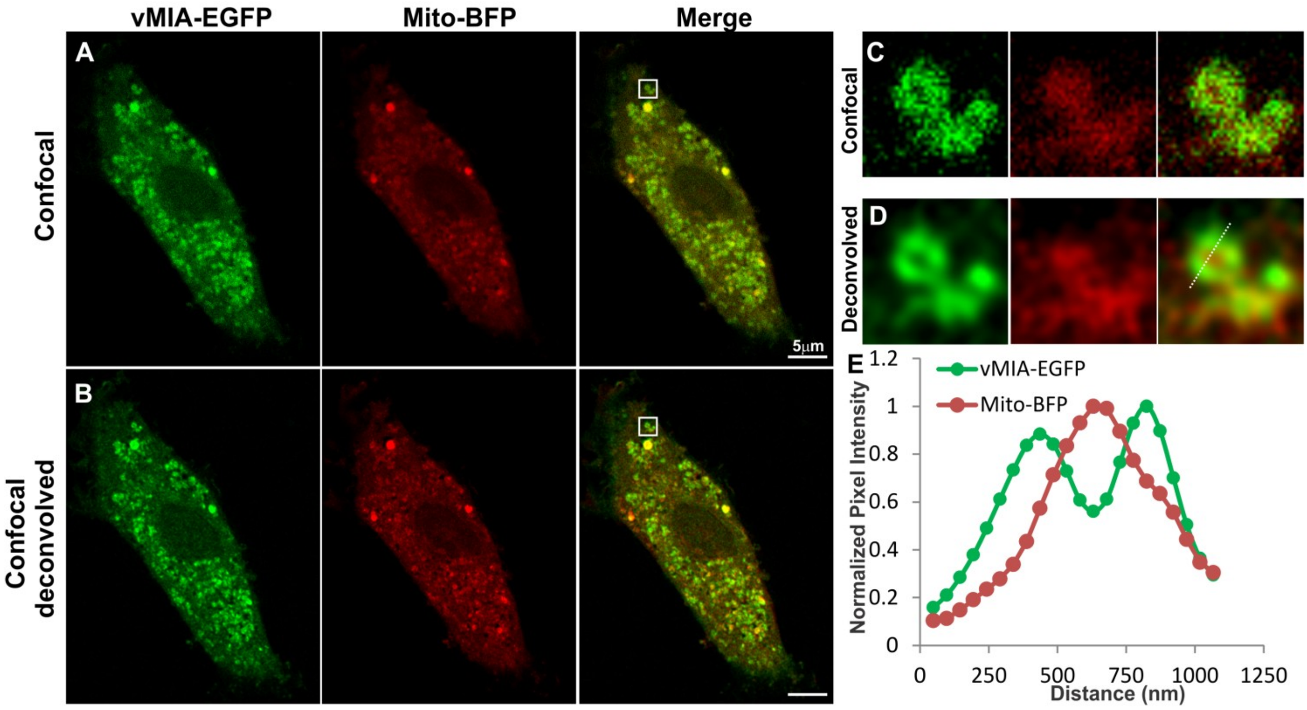

Supplemental Figure S2. Mitochondrial fragmentation by vMIA exhibits a threshold effect. Primary HFFs transfected with vectors to express vMIA-EGFP and Mito-BFP were fixed with 4% PFA at 22 hours after transfection as described in the methods and visualized by confocal deconvolution microscopy. (**A**) Maximal intensity projection of a 3-D image showing a pair of cells expressing differing levels of vMIA-EGFP (green), but comparable level of Mito-BFP (pseudocolored red). Insets showing the zoom of regions R1 and R2 highlights the change in mitochondrial morphology (as shown by Mito-BFP) in cells expressing low level (R1) or high level (R2) of vMIA-GFP (**B**) Maximal intensity 3-D projection of another cell expressing Mito-BFP (pseudocolored red in the merge) and showing varying levels of vMIA-EGFP (green in the merge) on the individual mitochondria. While most of the mitochondria in this cell have lost their tubular appearance, red arrows point to the individual mitochondrion that show low or no vMIA-GFP expression have remained tubular. Scale bars represent 5 μm.

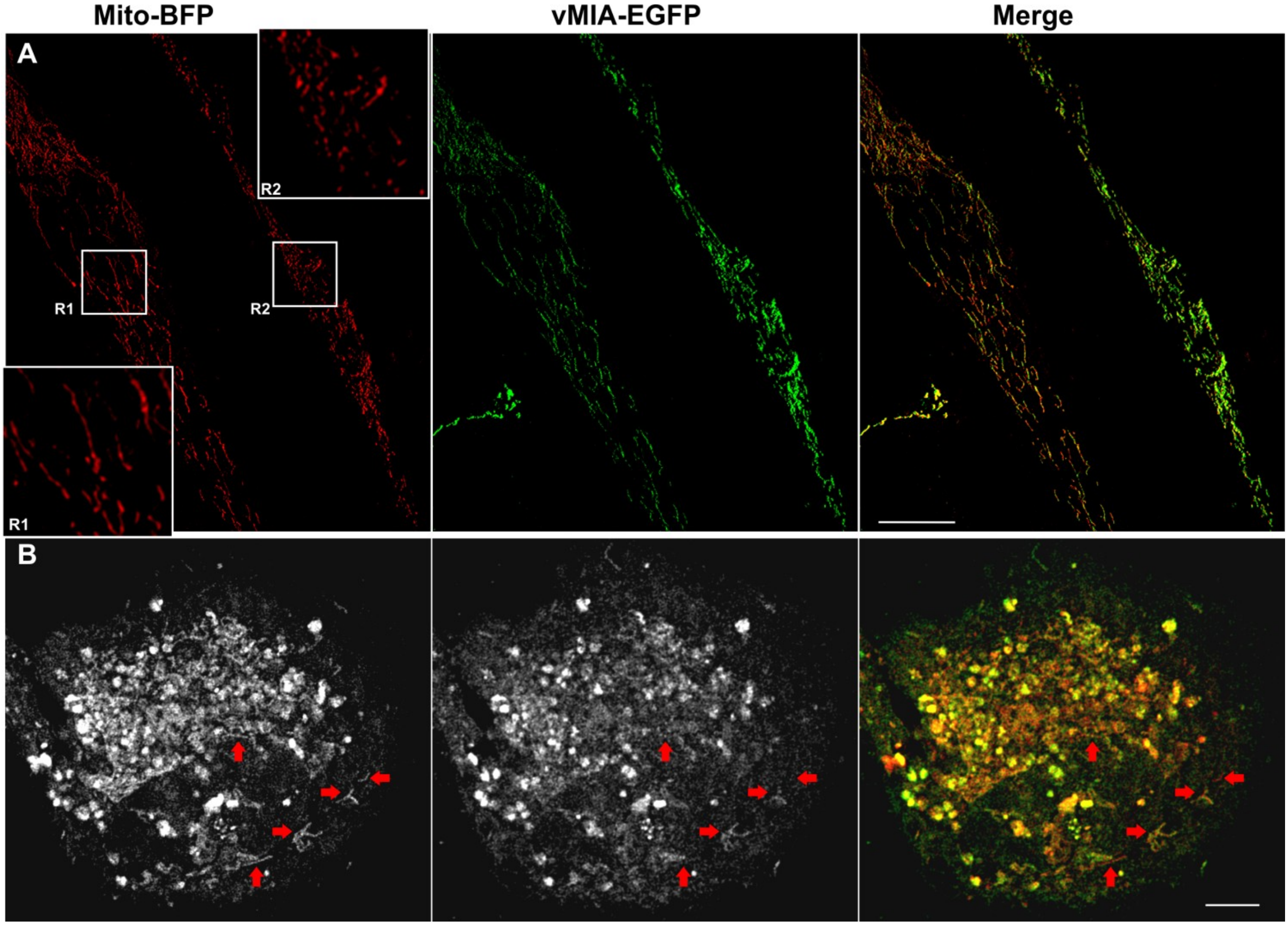

Supplemental Figure S3. Confocal microscopy imaging of ER-mitochondria interface. HFFs were lipofected with vectors expressing vMIA-EGFP (OMM), ss-RFP-KDEL (ER) and Mito-BFP (matrix) and fixed with 4% PFA at 25 hours after transfection as described below and previously published [23]. (**A**) Cells expressing vMIA-EGFP (green), ss-RFP-KDEL (red) and Mito-BFP (blue) were imaged using confocal microscopy. (**C**) The boxed region of interest from the merged image is enlarged. (**B**) Normalized intensity profiles of vMIA-EGFP (green), ss-RFP-KDEL (red) and Mito-BFP (blue) emissions are shown.

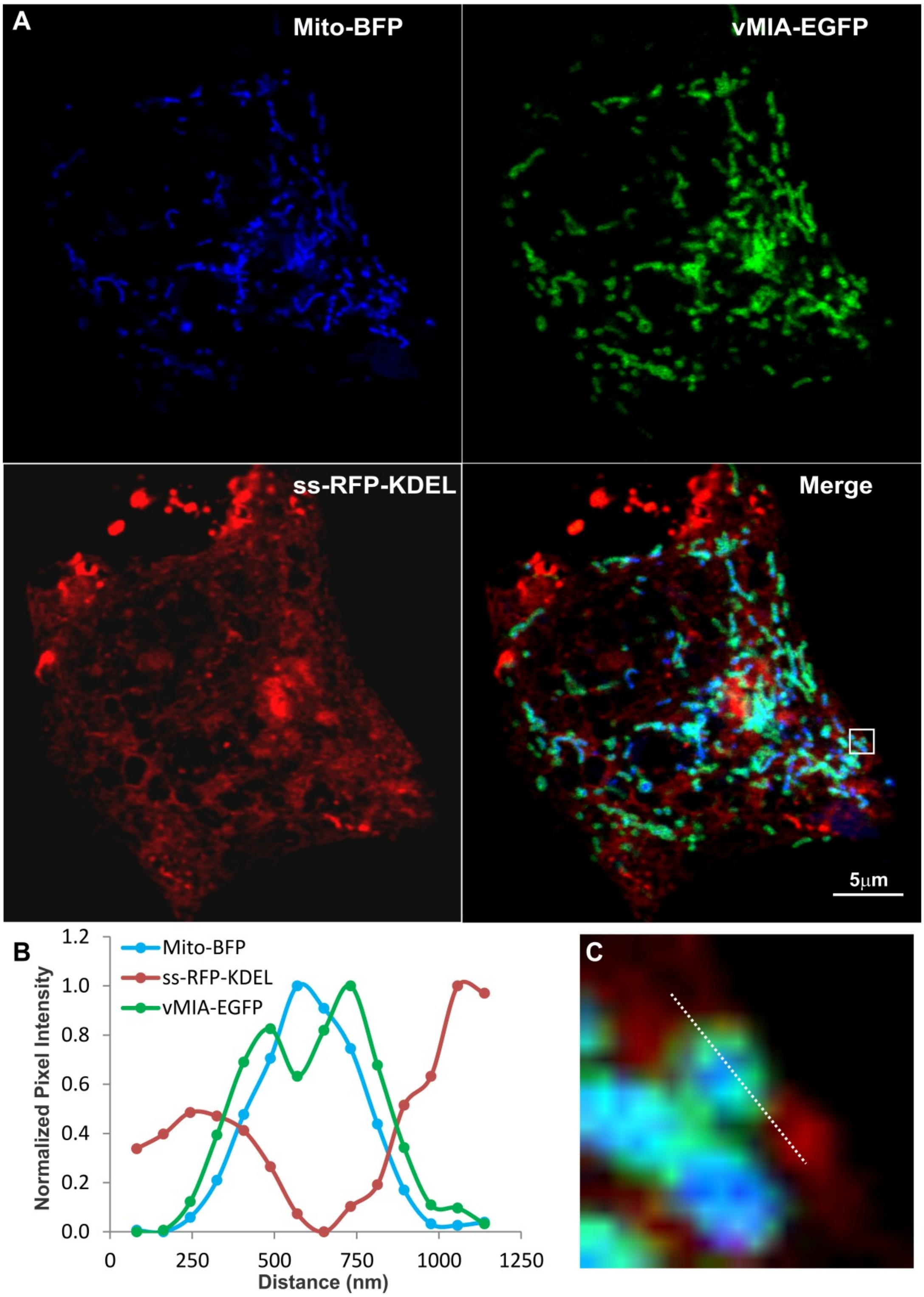

Supplemental Figure S4. GSTED microscopy of vMIA-EGFP in human fibroblasts. (**A**) HFFs were lipofected with vectors expressing vMIA-EGFP and DsRed-Mito. At 24 hours post transfection, cells were methanol fixed as described as below and published [23] and imaged using GSTED (vMIA-EGFP) and confocal microscopy (DsRed-Mito, vMIA-EGFP) followed by deconvolution of both the images. (**B**) The zoomed, merged image of a tubular mitochondrion in the boxed region (R1) is shown. This includes DsRed-Mito confocal (red), vMIA-EGFP confocal (blue) and vMIA-EGFP GSTED (green). Each channel is also presented individually. (**C**) Intensity profile of the pixels marked by the dotted line on the GSTED panel demonstrates the clustered distribution of vMIA along the entire length of the OMM of this mitochondrion. (**D**) The zoomed, merged image of a mitochondrion in the boxed region (R2) is shown. (**E**) The normalized intensity profile along the line shown on the R2 image, which demonstrates the significant improvement in visualizing the vMIA distribution along OMM by GSTED as compared to confocal imaging and its improved resolution of its localization away from the matrix and at the OMM.

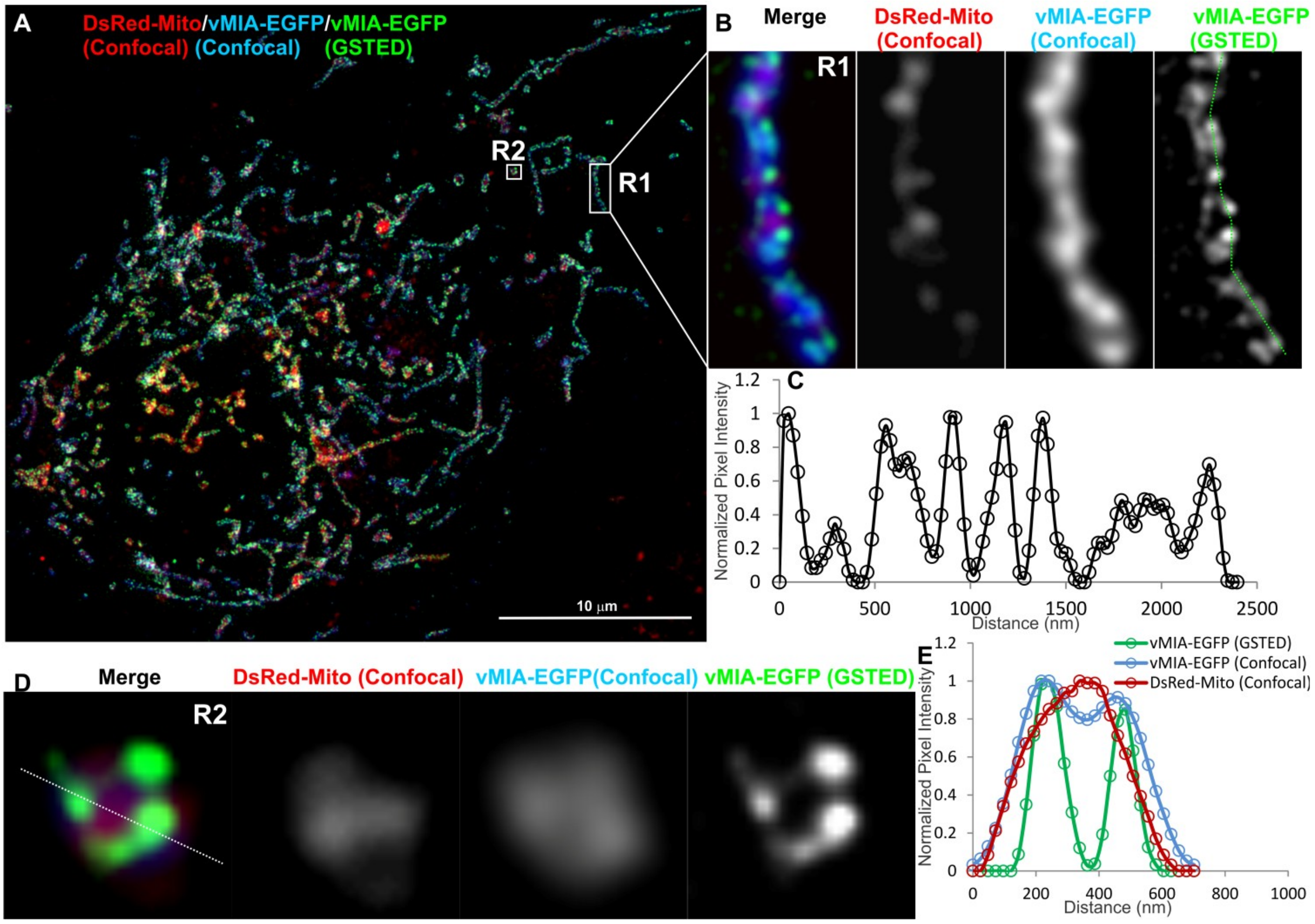

Supplemental Figure S5. GSTED microscopy of vMIA-EGFP in human astrocytoma cells. U373-TetOn expressing tTA [51] were lipofected with TRE-Tight promoter driving expression of vMIA-EGFP. At 24 hours post transfection, cells were treated with doxycycline for 60 minutes, labeled with MitoTracker Red (0.5 µM) and imaged live using Confocal (vMIA-GFP and MitoTracker Red) and GSTED (vMIA-GFP). (**A**) An optical slice showing deconvolved confocal image of a cell expressing vMIA-EGFP and a zoom of the region corresponding to the boxed region (R1) are shown in the left panel. Right panel presents the zoom of the R1 region showing the deconvolved GSTED (bottom) and deconvolved confocal MitoTracker (top) channels. (**B**) Zoomed confocal images of the mitochondrion in the region R2 showing the various channels acquired—Confocal images of MitoTracker Red and vMIA-EGFP as well as GSTED image of vMIA-EGFP. (**C**) Normalized intensity profile along the dotted line shown on the confocal and GSTED images of R2 demonstrate the improved resolution of vMIA localization by GSTED imaging as compared to confocal imaging. (**D**) Zoom of region R3 showing GSTED image of vMIA (**E**) Intensity profile of the pixels marked by the red line in panel (**D**).

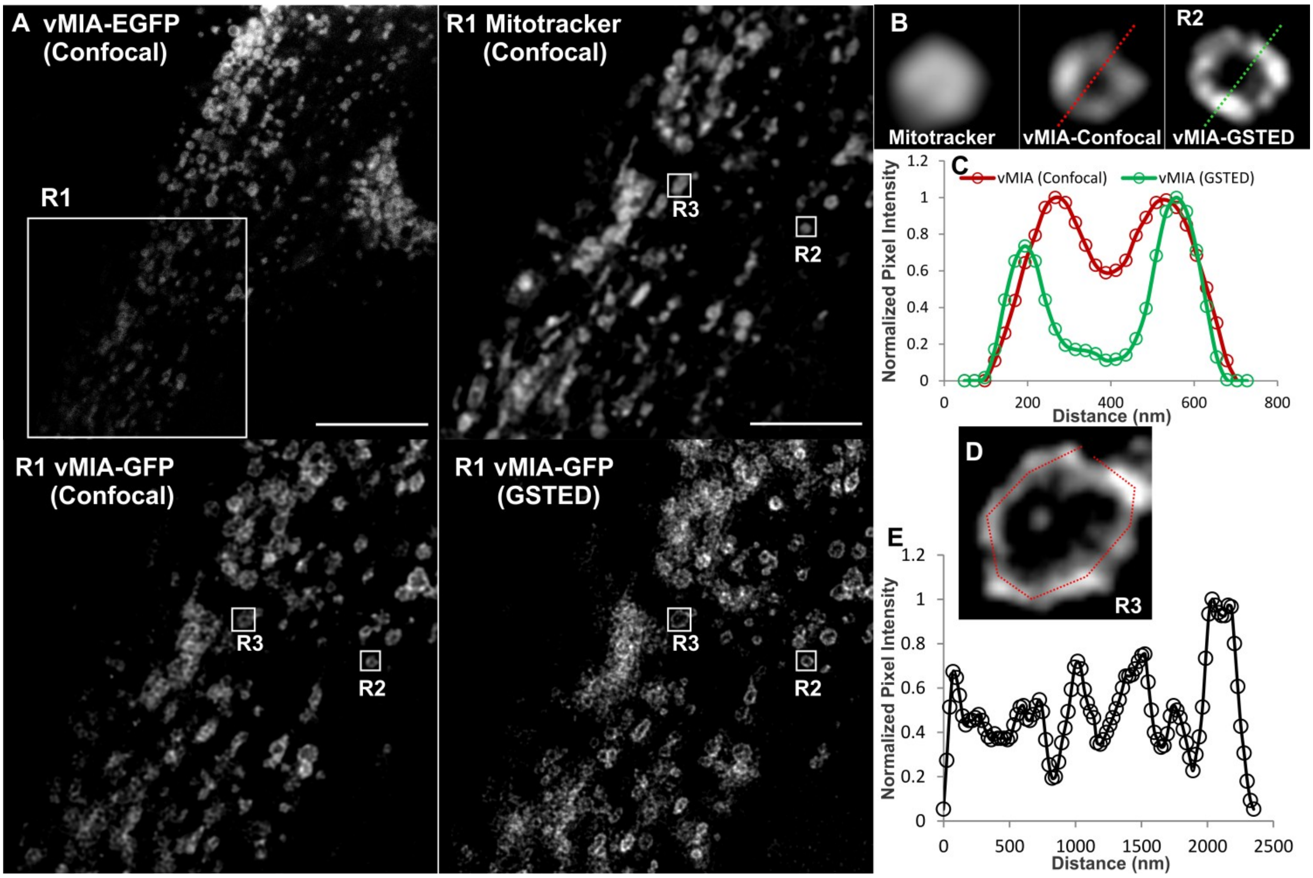

Supplemental Figure S6. Imaging vMIA localization using widefield microscopy and MSIM. HFFs were transfected with vectors expressing TRE-tight promoter-vMIA-EGFP (green) and its tetracycline controlled transactivator (tTA) [51]. Transfected cells were Dox-treated (0.2 µg/mL) for one hour, MitoTracker Red treated (0.5 µM for 5 min, red) and methanol fixed as described [23]. 25 slices (0.2 µm step size) were collected using 488 nm and 561 nm excitation lasers. For each channel, 256 multifocal excited images were collected as the excitation array was stepped ~1 pixel in a 16 × 16 grid pattern. (**A**) Summation of the images produces the widefield fluorescence image, which shows high degree of co-localization between vMIA-EGFP and MitoTracker Red. (**B**) Zoomed images of mitochondria in the region marked R3 are shown and the plot below shows the normalized intensity profile through the region marked by dotted line. (**C**) The MSIM image. (**D**) Zoomed images of region R3 and the normalized intensity profile for the region marked by dotted line show the improved ability to resolve the vMIA-EGFP and MitoTracker Red staining through the use of MSIM. (**E**) MSIM images of two mitochondria (R1 and R2). (**F**) Intensity profile through the perimeter of mitochondrion in R2 marked by dotted line. This indicates non-uniform distribution of vMIA-GFP along the mitochondrial membrane.

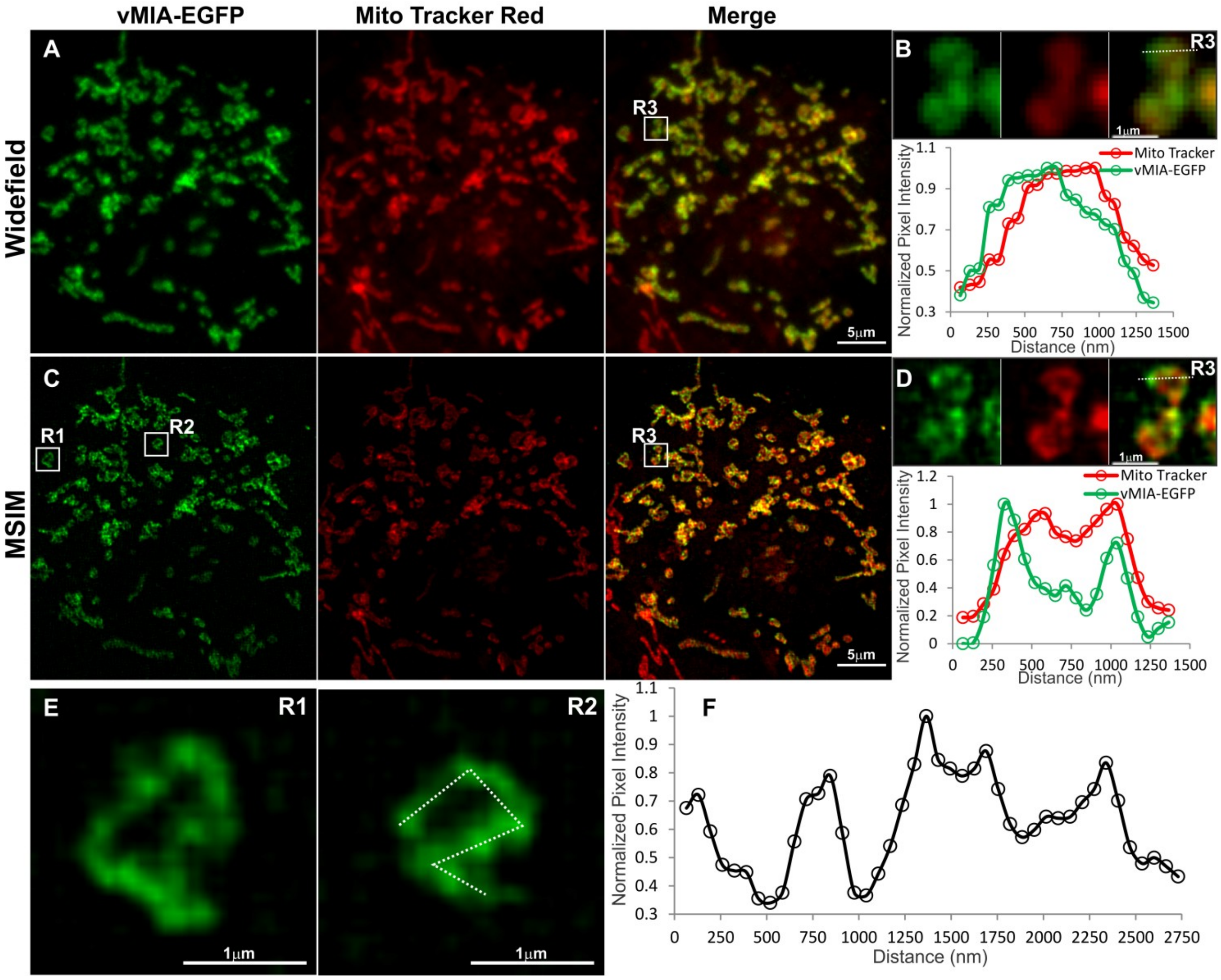

Supplemental Figure S7. PALM image of vMIA-PAmCherry. HFFs were transfected with vMIA-PAmCherry and imaged by PALM. The image in (a) shows 301666 molecules that were localized to ≤25 nm precision out of 515207. Molecules are plotted as two-dimensional Gaussian distributions with 25 nm standard deviations.

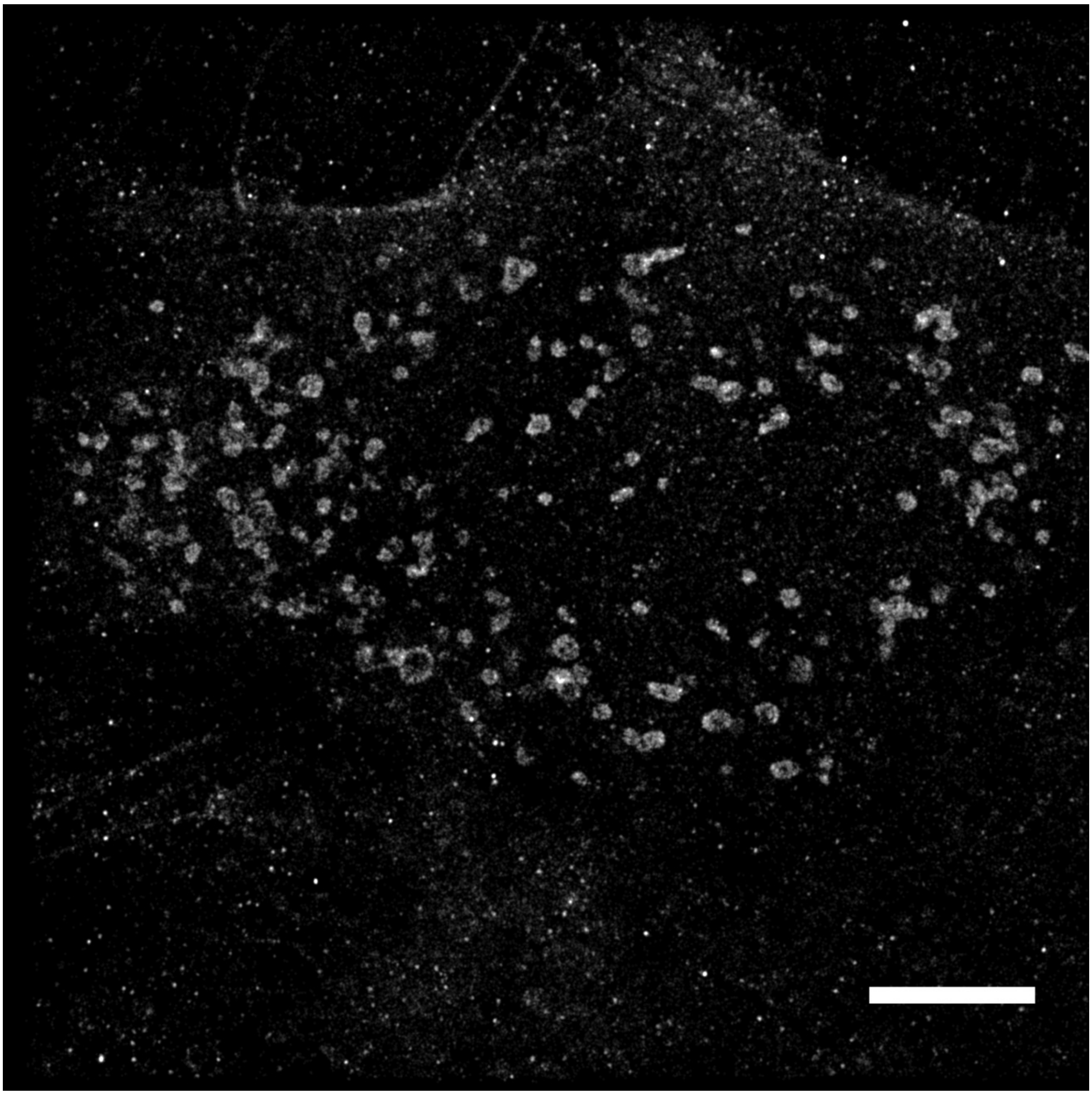

Supplemental Figure S8. Pair correlation analysis to test for molecule clustering. Pair correlation analysis requires determination of the particle density in a "shell" region around a reference particle (shown in green). The "shell" region is determined by the difference between two circular regions (r1 and r2) around the reference particle. The g(r) is the ratio of the particle density ρ within the δr region and the particle density ρ over the entire area if interest AN. Blue particles represent particles around the green reference particle which are not located in the δr region whereas the red particles are located inside δr.

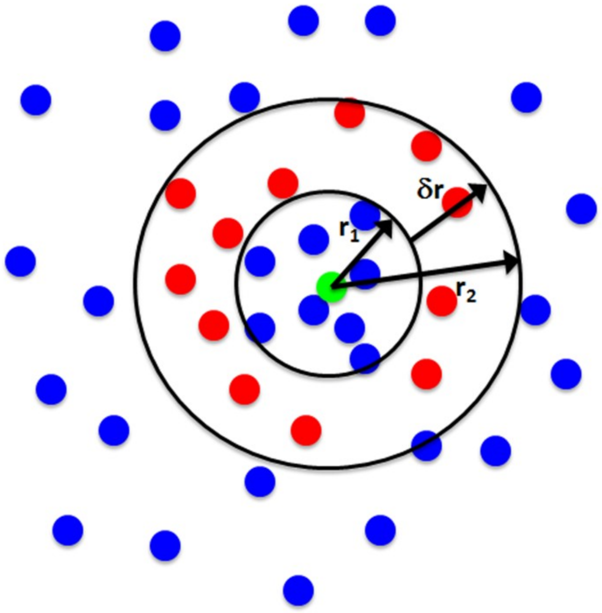

ρ=particle density

A_N=total number of particles considered

δr=difference between radius 1 and radius 2

$g(r) = \rho(\delta r)/\rho(A_N)$

Supplemental Figure S9. Demonstration and tests of pair correlation analysis using simulations of particle clustering. Images were simulated which contain 10,000 particles either (**A**) entirely randomly distributed, (**B**) partially clustered (~4 pixel radii), (**C**) partially clustered (~4 pixel radii) at twice the particle density, or (**D**) partially clustered at twice the particle density and twice the cluster radius (~9 pixel radii). (**E**) The g(r) was determined for each of these images and are designated as follows: random (green open circles), low density ~4 pixel clusters (red open squares), 2× density ~4 pixel clusters (blue filled circles), and 2× density ~9 pixel clusters (black filled squares).

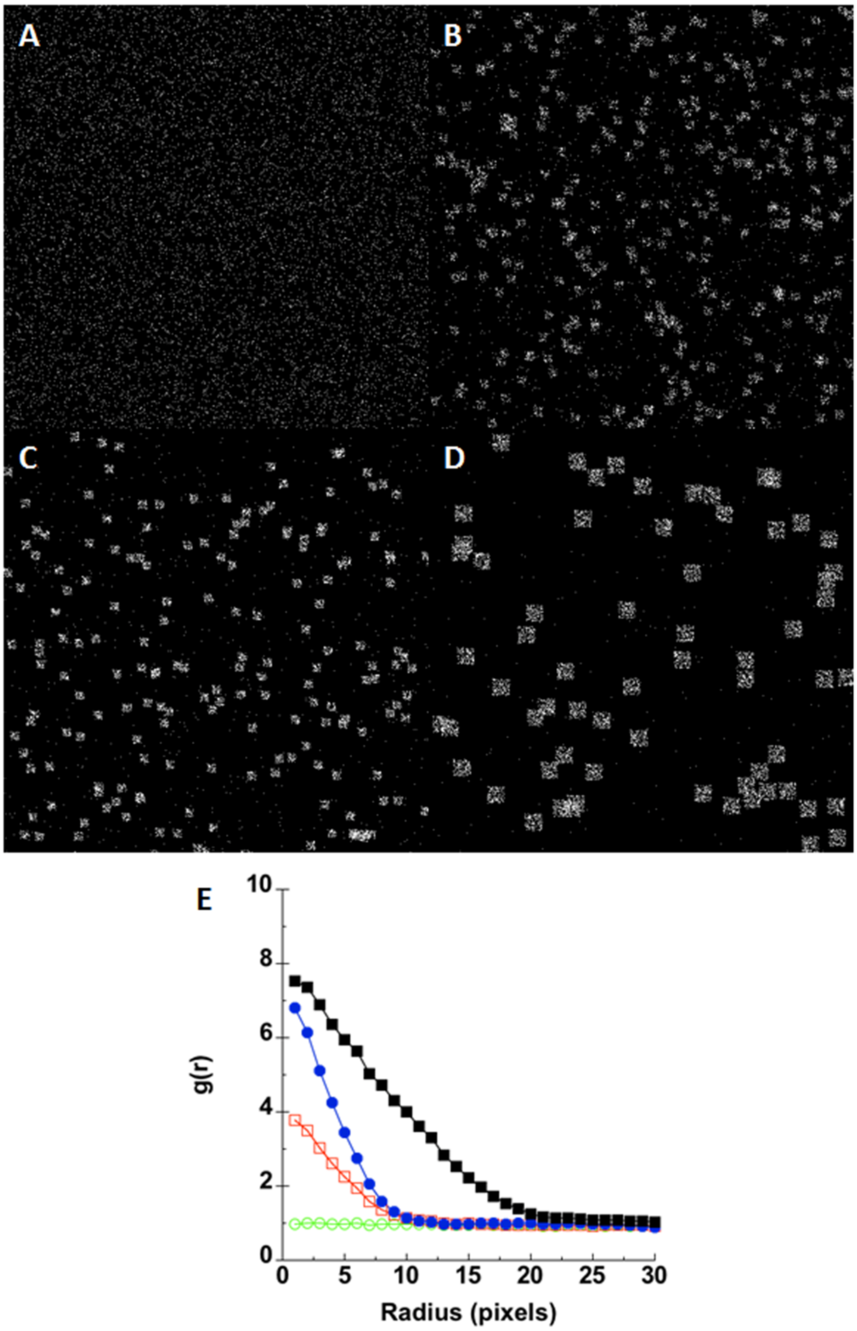

Supplemental Figure S10. Maximum g(r) from pair correlation analysis of PALM data. A binary image containing peak positions of 515269 molecules localized to ≤25 nm was subjected to paired correlation analysis. The g(r) for each molecule was determined and is plotted here with the pixel set with the maximum g value. The LUT and calibration bar are included in the upper right corner for reference. The scale bar is 5 μm.

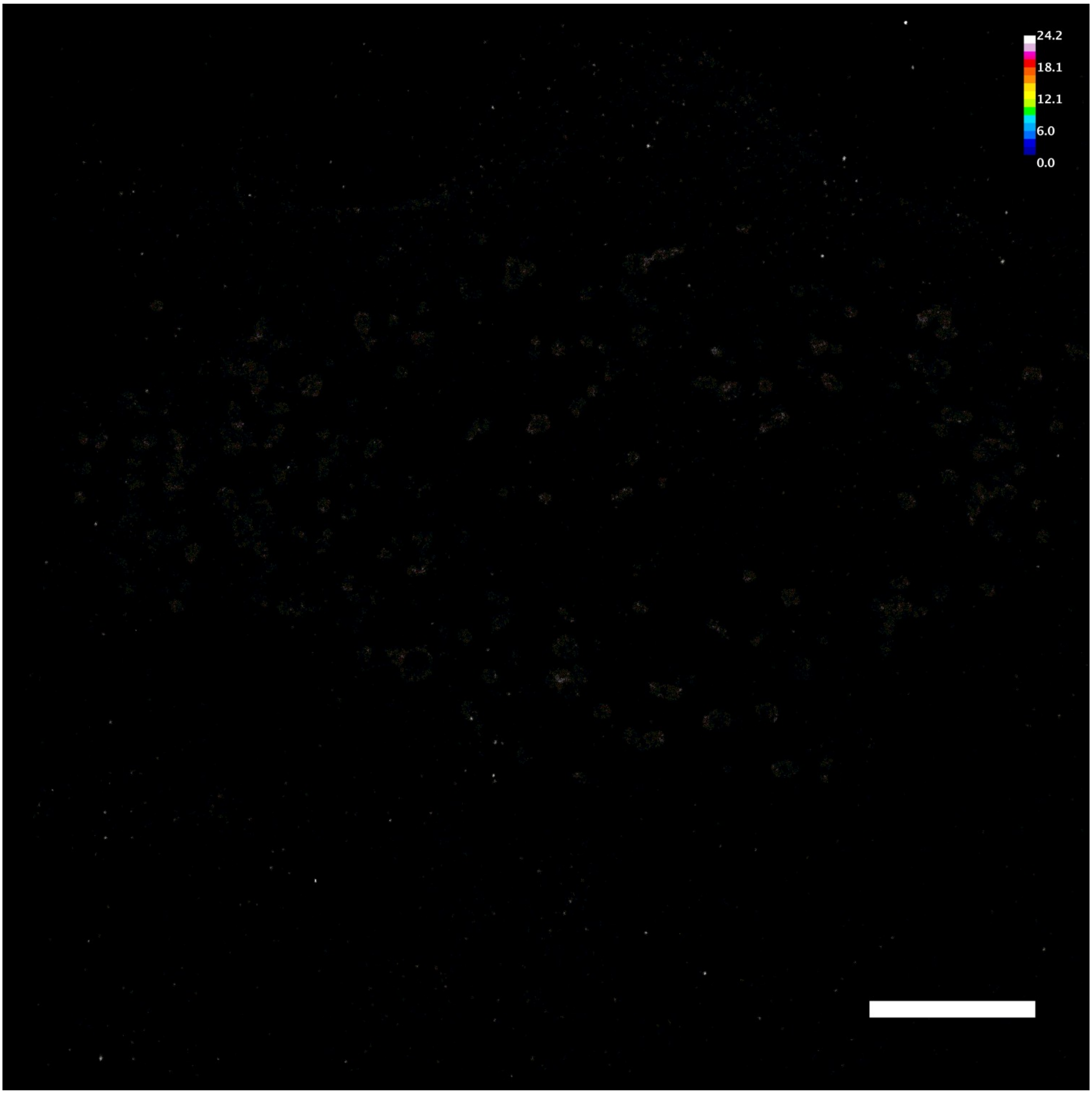

Supplemental Figure S11. Maximum g(r) from pair correlation analysis of PALM data rendered with 25 nm 2D Gaussian distributions. A binary image containing peak positions of 515269 molecules localized to ≤25 nm was subjected to pair correlation analysis. The g(r) for each molecule was determined and is plotted here with the pixel set with the maximum g value. The image was then processed with a Gaussian blur (sigma = 25 nm). The pixel values were scaled to match the LUT in Supplemental Figure S9. The LUT and calibration bar are included in the upper right corner for reference. The scale bar is 5 µm.

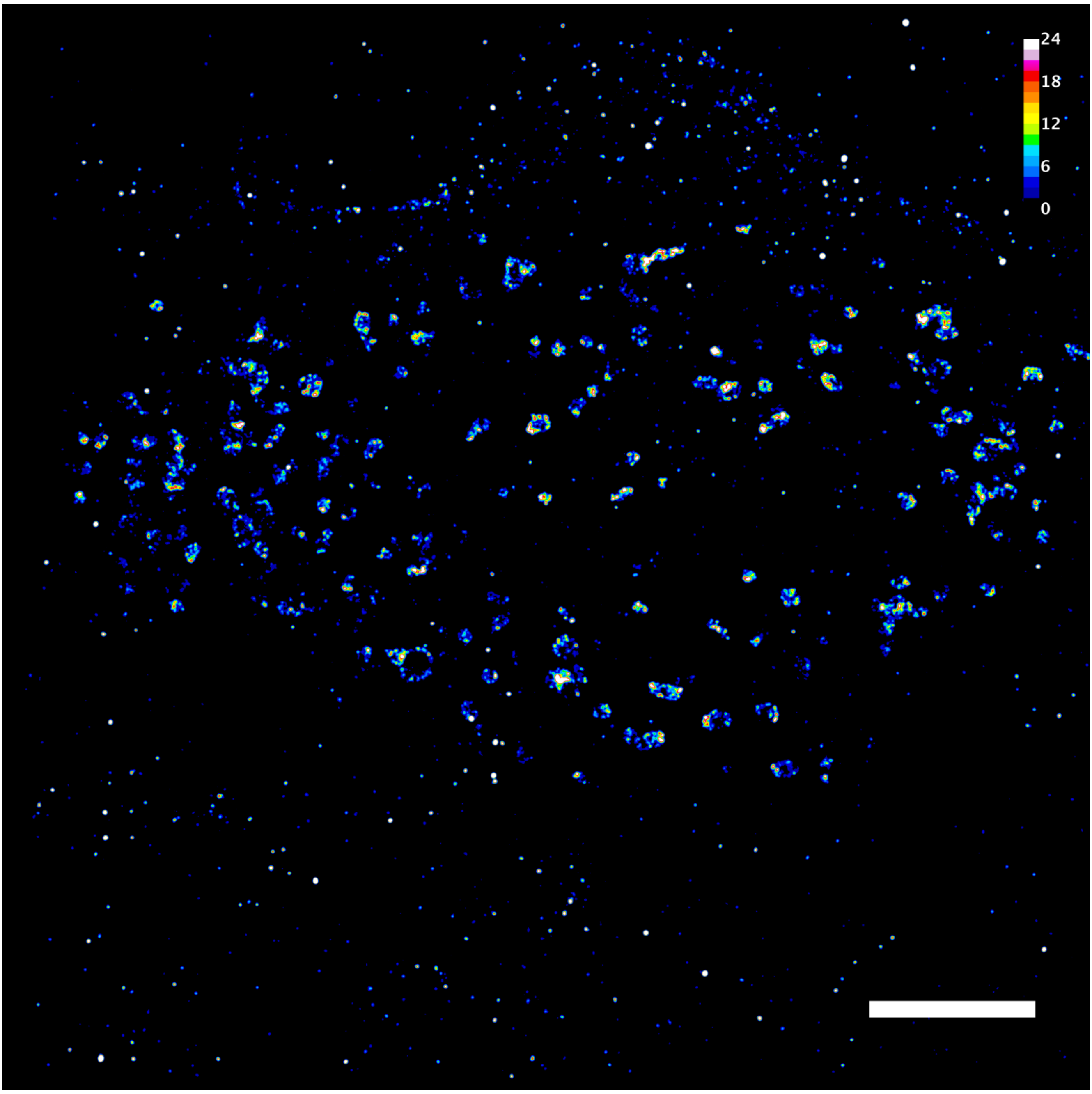

Supplement: Supplementary File 1 — Supplementary Information (PDF, 3325 KB) [file viruses-06-01612-s001.pdf]
